# Severity modeling of propionic acidemia using clinical and laboratory biomarkers

**DOI:** 10.1038/s41436-021-01173-2

**Published:** 2021-05-18

**Authors:** Oleg A. Shchelochkov, Irini Manoli, Paul Juneau, Jennifer L. Sloan, Susan Ferry, Jennifer Myles, Megan Schoenfeld, Alexandra Pass, Samantha McCoy, Carol Van Ryzin, Olivia Wenger, Mark Levin, Wadih Zein, Laryssa Huryn, Joseph Snow, Colby Chlebowski, Audrey Thurm, Jeffrey B. Kopp, Kong Y. Chen, Charles P. Venditti

**Affiliations:** 1 Medical Genomics and Metabolic Genetics Branch, National Human Genome Research Institute, National Institutes of Health (NIH), Bethesda, MD USA; 2 NIH Library, Office of Research Services, National Institutes of Health, Bethesda, MD USA; 3 Zimmerman Associates, Inc., Fairfax, VA USA; 4 Nutrition Department, Clinical Research Center, National Institutes of Health, Bethesda, MD USA; 5 New Leaf Center, Mount Eaton, OH USA; 6 National Heart Lung and Blood Institute, National Institutes of Health, Bethesda, MD USA; 7 Ophthalmic Genetics and Visual Function Branch, National Eye Institute, National Institutes of Health, Bethesda, MD USA; 8 National Institute of Mental Health, National Institutes of Health, Bethesda, MD USA; 9 National Institute of Diabetes and Digestive and Kidney Diseases, National Institutes of Health, Bethesda, MD USA

## Abstract

**Purpose:**

To conduct a proof-of-principle study to identify subtypes of propionic acidemia (PA) and associated biomarkers.

**Methods:**

Data from a clinically diverse PA patient population (https://clinicaltrials.gov/ct2/show/NCT02890342) were used to train and test machine learning models, identify PA-relevant biomarkers, and perform validation analysis using data from liver-transplanted participants. *k*-Means clustering was used to test for the existence of PA subtypes. Expert knowledge was used to define PA subtypes (mild and severe). Given expert classification, supervised machine learning (support vector machine with a polynomial kernel, svmPoly) performed dimensional reduction to define relevant features of each PA subtype.

**Results:**

Forty participants enrolled in the study; five underwent liver transplant. Analysis with *k*-means clustering indicated that several PA subtypes may exist on the biochemical continuum. The conventional PA biomarkers, plasma total 2-methylctirate and propionylcarnitine, were not statistically significantly different between nontransplanted and transplanted participants motivating us to search for other biomarkers. Unbiased dimensional reduction using svmPoly revealed that plasma transthyretin, alanine:serine ratio, GDF15, FGF21, and in vivo 1-^13^C-propionate oxidation, play roles in defining PA subtypes.

**Conclusion:**

Support vector machine prioritized biomarkers that helped classify propionic acidemia patients according to severity subtypes, with important ramifications for future clinical trials and management of PA.

**Graphical Abstract:**

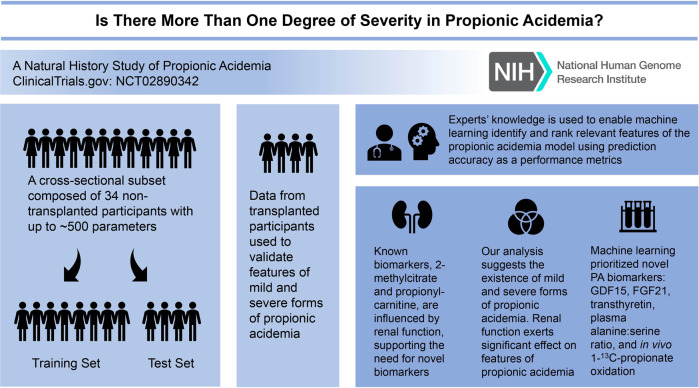

## INTRODUCTION

Propionic acidemia (PA) is an organic acidemia caused by the reduced activity of propionyl-CoA carboxylase (PCC) due to pathogenic variants in *PCCA* and *PCCB*. PA is characterized by accumulation of propionyl-CoA-derived metabolites, leading to episodes of metabolic acidosis and multiple organ damage. Affected patients display a range of clinical severity.^1–4^ The existence of a mild PA phenotype was suggested more than 40 years ago with the description of a “Mennonite Amish” variant of PA, eventually linked to a homozygous c.1606A>G (p.Asn536Asp) variant in *PCCB*.^5,6^ However, what constitutes mild versus more severe types of PA, their defining features, such as other genotypes and biomarkers associated with them, has not been established. Propionylcarnitine (C3) and 2-methylcitrate (2-MC) are widely used diagnostic biomarkers to screen presymptomatic newborns for disorders of propionate metabolism,^7,8^ yet their usefulness to predict the natural history of PA is incompletely understood.^4^ Liver transplantation in PA aiming to restore hepatic oxidation of propionate created an opportunity to evaluate the performance of C3 and 2-MC pharmacodynamic (response) and, potentially, as surrogate biomarkers. Surprisingly, several studies of the long-term outcomes of liver transplantation in PA revealed that C3 levels before and after the transplant were not significantly different,^9–11^ prompting questions whether these biomarkers can serve as pharmacodynamic (response) or surrogate biomarkers for PA in a treatment trial.

Identifying biomarkers with predictive characteristics has ramifications for the design of proposed genomic treatments, including messenger RNA (mRNA and adeno-associated virus–based gene therapy.^12^ The ability to prognosticate based on biomarker data depends on (1) the availability of a genetically diverse cohort to model the spectrum of PA, (2) sufficient time to track changes in individual patients, and (3) longitudinal measurements of relevant clinical and laboratory parameters. Advances in data science can facilitate identification of predictive biomarkers in an objective fashion, especially in rare diseases like PA, where tests with many dimensions are routinely measured, such as amino acid and acylcarnitine profiles. However, analyses of multidimensional datasets are difficult due to the challenges of identifying complex patterns among categorical and continuous variables that often influence each other nonlinearly.^13^

Multidimensional phenotyping of a rare disease often leads to datasets characterized by many predictors (*p*) and few participants (*n*) (*p* >> *n*). This can lead to effect sparsity, i.e., a condition where only some in the large set of predictors actually influence the outcome of interest.^14^ Support vector machine methods are well suited as “out of the box” solutions for analytical tasks requiring classification and dimensional reduction.^15^ However, when applied to the field of inborn errors of metabolism, the utility of machine learning has been primarily demonstrated in improving the accuracy of newborn screen calls in datasets composed of hundreds of thousands of participants and a relatively few variables (*n* > *p*).^16–18^

Using a relatively small but multidimensional dataset of participants with PA studied through a dedicated natural history protocol (*n* = 40, *p* ~ 500), we demonstrate how expert knowledge augmented by machine learning can reveal the existence of clinically relevant subtypes of PA and associated biomarkers. This proof-of-principle paradigm may be extended to other rare diseases, where the number of participants is small, but disease classification is crucial, and discovery of biomarkers is required.

## MATERIALS AND METHODS

### Definition of outcomes and study design

The primary outcome of interest was to identify classes of propionic acidemia based on the severity of their manifestations and variables associated with each class. A secondary outcome of interest was to identify novel laboratory parameters that can help ascertain classes and serve as candidate pharmacodynamic/response biomarkers or surrogate endpoints. To achieve these outcomes, we used data from a cross-sectional dataset generated through a dedicated natural history study. The cohort of 40 patients was divided into three cohorts: dynamically generated training and test sets composed of nontransplanted PA participants and a validation group (*n* = 5) composed of participants after a liver transplant. Candidate pharmacodynamic/response biomarkers or surrogate endpoints were identified using severity assignments of three experts augmented using a machine learning algorithm, support vector machine with a polynomial kernel (svmPoly) (See Visual Summary).

### Patient cohort and clinical protocols

The clinical research protocol (ClinicalTrials.gov identifier: NCT02890342) was approved by the National Institutes of Health Institutional Review Board prior to the initiation of the study. PA was confirmed through clinical, biochemical, and/or molecular evaluation (Supplemental Table 1). Forty participants enrolled in the study; five underwent liver transplant. Clinical, laboratory, and genetic parameters were studied on all protocol participants (See Supplemental Materials and Methods for more details).

Baseline CO_2_ production (VCO_2_) was measured using indirect calorimetry during a resting energy expenditure study.^19^ 1-^13^C-propionate was administered orally or as bolus via a gastric or jejunal tube. Breath samples were collected serially. Isotope ratio mass spectrometry (IRMS, ^13^C/^12^C) of CO_2_ in the expired breath was used to determine the percent ^13^CO_2_ recovery over 2 hours (at 30, 60, 120 minutes), and with baseline CO_2_ production rate, used to calculate propionate oxidation capacity.^19,20^

### Dataset and statistical analysis

The cross-sectional dataset included 40 study subjects, with up to 483 variables ascertained per participant. For participants seen more than once at the NIH Clinical Research Center, the most recent and most complete data were used. Statistical analysis was performed using either GraphPad Prism 8 (GraphPad Software, San Diego, CA) or R (R Foundation for Statistical Computing, Vienna, Austria).^21^

For machine learning analysis, variables with missing data for >70% of participants were removed; this represented 172 variables. We retained 301 variables. Missing data were imputed using the *k*-nearest neighbors algorithm (R package VIM, a kNN method)^22^ and Euclidean distances were normalized using a min–max procedure (a linear transformation of *x* to *y* = [*x*–min]/[max–min]).

The difference in means or medians between two groups was detected using a paired or unpaired Student *t*-test for normally distributed data and using a Mann–Whitney *U* test for non-normally distributed data. The comparison of means or medians for three or more groups was assessed using a one-way analysis of variance (ANOVA) followed by Tukey’s multiple comparisons test. If the data did not support the assumption of normality, the Kruskal–Wallis test was used followed by the Dunn’s multiple comparisons test. The association between two variables was assessed using a simple linear regression for continuous variables and logistic regression for categorical variables. A *p* value *<0.05 was interpreted as statistically significant. Further distinctions in statistical significance were denoted by **p < 0.01, ***p < 0.001, ****p < 0.0001.

### Machine learning

To help construct a severity model of PA, 14 clinical and laboratory variables were selected based on their potential to describe the biochemical severity and multiorgan involvement in PA: plasma C3 (umol/L), plasma total 2-MC (nmol/L), full-scale IQ (FSIQ), optic nerve abnormality (absent = 0, present = 1), sensorineural hearing loss (absent = 0, present = 1), height *z*-score, alanine aminotransferase (ALT, units per liter), peripheral white blood cell count (WBC, k/uL), peripheral red blood cell count (RBC, m/uL), peripheral platelet count (k/uL), left ventricular ejection fraction (LVEF%), cystatin C–based estimated glomerular filtration rate (eGFR mL/min/1.73 m^2^), total protein intake (% of recommended dietary allowance), and incomplete protein intake (% of recommended dietary allowance).

To establish a plausible number of possible clusters in the dataset, which would represent classes of propionic acidemia, we used an unsupervised machine learning approach using *k*-means clustering. An elbow plot was used to identify the number of possible PA classes. Supervised machine learning was carried out using support vector machine (SVM) with a polynomial kernel function (svmPoly, R package “caret”).^23^ svmPoly was also used to measure the influence (variable importance) of each parameter on the SVM model of PA.

To enable machine learning using SVM, we first defined the PA disease model as a dimensional space composed of 14 clinical continuous and categorical variables (Supplemental Fig. 1, Supplemental Materials and Methods). A mild or severe PA class was assigned to each participant by three judges with expert knowledge of the natural history of organic acidemias. Each judge was provided with standardized clinical summaries of all nontransplanted PA participants and was asked to classify them as a mild or severe type of PA. Each judge was able to review clinical and laboratory parameters in the NIH electronic medical records, excluding novel biomarkers. Class assignments were then tallied and in cases when judges disagreed in the assigned class, a simple majority was determinative. Interrater agreement analysis is shown in Supplemental Table 3.

As a comparison, we assessed the accuracy of a prototype of the organic acidemia severity scale, originally developed by Walter et al. and updated by Thompson et al. (Supplemental Materials and Methods).^24,25^

The main metric of each SVM model performance was accuracy, defined as Accuracy = (TP + TN) / (TP + TN + FP + FN), where TP is the number of true positive, TN is the number of true negative, FP is the number of false positive, and FN is the number of false negative prediction. Each svmPoly model was ranked based on its accuracy in predicting judges’ score to identify a model composed of the minimum set of parameters (dimensional reduction) with the highest accuracy (Supplemental Fig. 2). Established and novel biomarkers prioritized by the svmPoly model underwent validation analysis using a dataset composed of liver-transplanted PA participants (*n* = 5).

## RESULTS

### Plasma total 2-MC and C3 are affected by declining eGFR

**Fig. 1:**
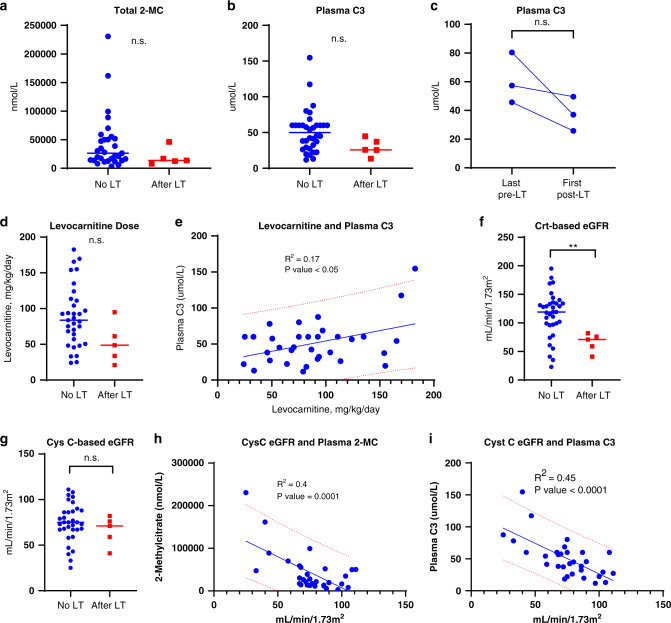
Propionic acidemia (PA) biomarkers, plasma propionylcarnitine and plasma 2-methylcitrate, are associated with levocarnitine dose and eGFR. (**a**,**b**) There was no difference in the levels of total plasma 2-methylcitrate (2-MC) and propionylcarnitine (C3) between unpaired transplanted and nontransplanted cohorts. (**c**) There was no difference in the C3 levels between paired pre- and post–liver transplant samples. (**d**) Enteral carnitine dosage was not different between transplanted and nontransplanted participants. (**e**) Plasma C3 and a levocarnitine dosage showed a significant, but modest, association. (**f**–**g**) Creatinine-based eGFR was lower in liver-transplanted participants. (**h**–**i**) Plasma 2MC and C3 showed significant association with cystatin C–based eGFR. Significant *p* values are denoted by *p < 0.05, **p < 0.01, ***p < 0.001, ****p < 0.0001. 2-MC 2-methylcitrate, C3 propionylcarnitine, Crt-based eGFR creatinine-based estimated glomerular filtration rate, Cys C-based eGFR cystatin C–based estimated glomerular filtration rate, LT liver transplant, n.s. not significant.

We reasoned that PA participants who received liver transplants may acquire some traits of a biochemically milder form of PA, which can help determine the utility of plasma total 2-MC and C3 to discern the effects of improved propionate metabolism on novel PA biomarkers (Supplemental Fig. 4). We first compared established PA biomarkers, plasma total 2-MC, C3, and other parameters (e.g., eGFR) that can affect these biomarkers, in participants with and without liver transplants. Although there was a trend toward statistical significance (*p* = 0.13 for 2-MC and *p* = 0.06 for C3), there was much overlap in values between each group (Fig. 1a, b). We did not detect a difference between available paired pre- and post–liver transplant plasma C3 samples (Fig. 1c). Pretransplant 2-MC was not available. We found no difference in the dosing of levocarnitine between nontransplanted and transplanted participants (Fig. 1d). A statistically significant, but weak, association existed between levocarnitine dose and plasma C3 (Fig. 1e, *r* = 0.41, *p* < 0.05). Transplanted participants had a statistically significantly lower creatinine-based, but not cystatin C–based eGFR values (Fig. 1f, g). Association between the estimated eGFR and plasma total 2-MC and C3 was present (Fig. 1h, i; data for creatinine-based eGFR not shown).

Collectively, these data revealed the effect of levocarnitine on plasma C3 and total 2-MC, as well as associations with eGFR. This may offer a partial explanation (adjusted *R*^*2*^ = 0.43) for why these biomarkers underperformed as response biomarkers and were not significant for liver-transplanted participants. However, we could not exclude the possibility that the inherent severity of PA may also influence plasma C3 and total 2-MC levels. To evaluate the role of PA severity, we conducted a series of machine learning experiments to reveal classes of PA and variables associated with them.

### *k*-Means clustering suggested the existence of several PA classes

**Fig. 2:**
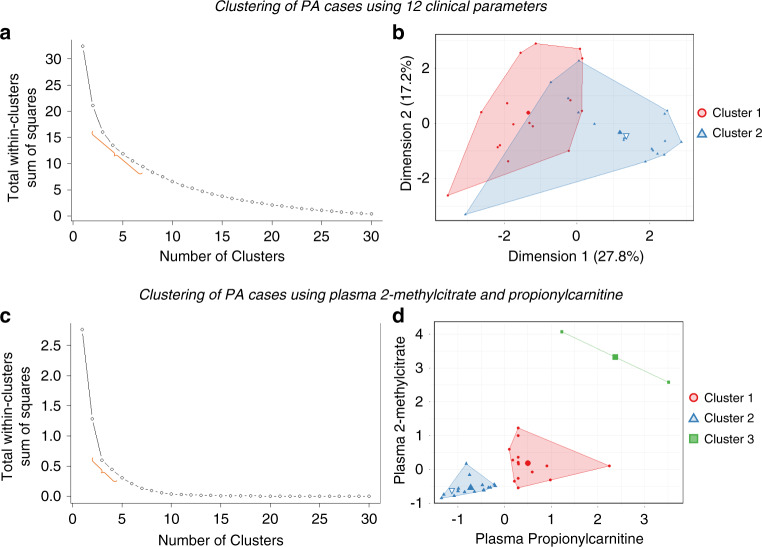
*k*-Means clustering suggested the existence of two or more classes of propionic acidemia (PA). (**a**) The elbow method of clustering analysis of 12 clinical parameters (denoted by an orange brace) suggested the existence of several classes of PA. Since the low number of participants in each group adversely affected our ability to perform supervised machine learning, in all subsequent analysis we posited two classes of PA (mild and severe). (**b**) *k*-Means clustering of 12 clinical parameters using two centroids (shown as a large filled blue triangle and red circle) did not unequivocally separate the classes of PA. An inverted triangle with white color fill denotes a patient homozygous for two Amish alleles (*PCCB*: c.1606A>G; p.Asn536Asp). (**c**) The elbow analysis on the two known biochemical parameters (plasma propionylcarnitine and total plasma 2-methylcitrate) suggested the existence several classes of propionic acidemia. (**d**) *k*-Means clustering analysis of the two biochemical parameters using three centers suggested the existence of three classes of PA. An inverted triangle with white color fill denotes a patient homozygous for two Amish *PCCB* alleles (*PCCB*: c.1606A>G; p.Asn536Asp). Cluster 3 was composed of two participants whose clinical course was characterized by progressive chronic kidney disease and a low left ventricular ejection fraction. Better separation of clusters using biochemical parameters motivated our search for a novel biochemical biomarker, which would be less affected by the renal status.

After defining a PA disease model using 12 clinical and 2 biochemical parameters (Supplemental Fig. 1), we proceeded to identify *k*-clusters among non-liver-transplanted participants. *k*-Means clustering using only clinical parameters did not achieve a nonoverlapping partition of participants (a two-cluster model shown in Fig. 2a, b, Hopkins statistic for cluster tendency = 0.65). *k*-Means clustering using two biochemical parameters achieved partitioning (a three-cluster model shown in Fig. 2c, d, Hopkins statistic = 0.86) with a pattern of grouping based on the concentration of plasma total 2-MC and C3. As expected, a participant homozygous for the previously described “Amish” allele (*PCCB*:c.1606A>G, p.Asn536Asp) segregated within a cluster with the lowest levels of plasma total 2-MC and C3 (denoted by an inverted blue triangle within cluster 1 in Fig. 2b, d). Cluster 3 (denoted by green colored squares in Fig. 2d) was composed of two participants whose clinical course included substantial renal involvement, supporting the notion that advanced kidney disease in PA patients constitutes a particular PA subtype. After determining that there could exist two or more subtypes of PA along a biochemical continuum of manifestations, we proceeded to explore clinical and biochemical parameters, or a combination of thereof, that might define these subtypes.

### Supervised machine learning can aid in the unbiased selection of novel PA biomarkers

We noted the small number of participants in some classes identified by unsupervised machine learning (e.g., only two participants in cluster 3 as in Fig. 2c, d) and faced the need to increase the variance in parameter spaces to enable supervised machine learning. For this, we used judges’ class assignments of each participant as mild or severe and analyzed clinical and laboratory parameters for their association with a given class (Supplemental Table 4).

**Fig. 3:**
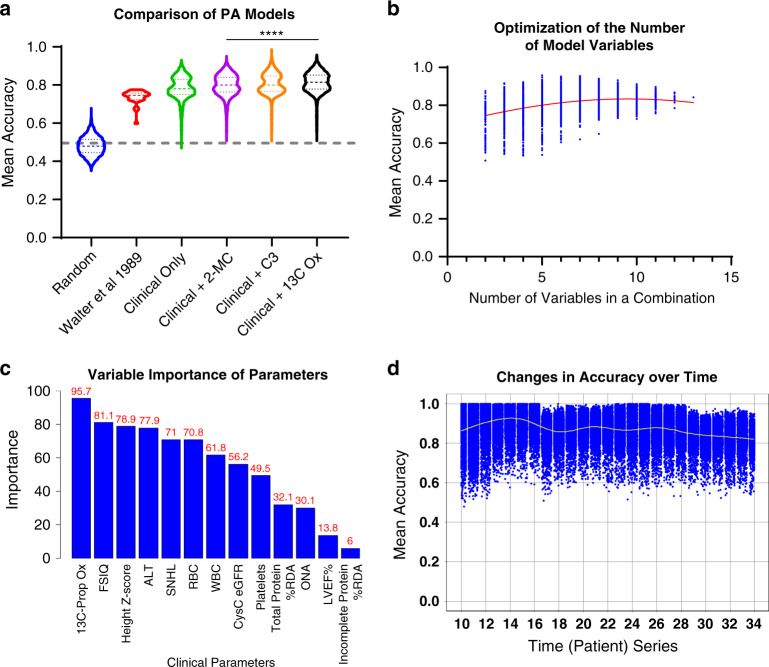
Support vector machine modeling defines the role of propionylcarnitine and total 2-methylcitrate and identified novel biomarkers delineating classes of propionic acidemia (PA). (**a**) Comparison of mean accuracies generated by support vector machine models using 12 clinical parameters versus 12 clinical parameters plus 1 biochemical variable (2-MC, C3, or 1-^13^C-propionate oxidation). The support vector machine model using 12 clinical parameters plus 1-^13^C-propionate oxidation had the highest mean accuracy in predicting judges’ scores than any other model. (**b**) The number of variables in each combination affects the mean accuracy of model. Each dot represents the mean accuracy of 1,000 resamplings of the order in which participants could have joined the study. This figure demonstrates that the overall performance of models including 12 clinical parameters plus 1-^13^C-propionate oxidation peaks around 6–10 variables per combination. (**c**) Averaged variable importance of parameters in the final support vector machine model using 12 clinical variable plus 1-^13^C-propionate oxidation generated from 1,000 random resamplings. This statistical experiment revealed that the model prioritized 1-^13^C-propionate oxidation, FSIQ, height *z*-score as the top three variables. (**d**) The time-ordered series composed of the order in which PA participants joined the study suggests that model accuracy plateaued after the first ten participants. Degradation of the mean accuracy coincided with when a participant was added, whose PA class did not reach the consensus among judges (e.g., after subject 17). ^13^C Ox 1-^13^C-propionate oxidation, 2-MC total 2-methylcitrate, C3 propionylcarnitine, FSIQ full scale IQ, LT liver transplant, n.s. not significant. ALT alanine aminotransferase, SNHL sensorineural hearing loss, RBC red blood cells, WBC white blood cells, CysC eGFR cystatin C-based eGFR, LVEF%, percent ejection fraction of left ventricle, %RDA recommended dietary allowance, ONA optic nerve abnormality. Significant *p* values are denoted by *p < 0.05, **p < 0.01, ***p < 0.001, ****p < 0.0001.

We first tested the prediction accuracy using randomly generated classes, mild or severe, assigned to each participant. As expected with the random class assignment, the mean prediction accuracy was 0.5 (Fig. 3a). We then tested a prototype of the organic acidemia severity scale, developed by Walter et al. to establish its association with chronic kidney disease (CKD), one of the factors contributing to morbidity and mortality in isolated methylmalonic acidemia.^25^ The mean accuracy of prediction models based on the Walter severity scale was 0.74 with the best model’s accuracy reaching 0.77 (Supplemental Table 5).

We next used 12 clinical PA parameters (Fig. 3a, excluding plasma total 2-MC or C3). The mean accuracy of all possible svmPoly models was 0.78. The combination with the highest mean accuracy (0.93, mean sensitivity 0.94, mean specificity 0.92) included five parameters, FSIQ, ALT, WBC, RBC, and cystatin C-base eGFR (Supplemental Table 5). To evaluate the effects of established PA biomarkers on the mean accuracy, we ran models on 12 clinical parameters plus 1 parameter (e.g., 12 clinical parameters plus 2-MC or 12 clinical parameters plus C3, Fig. 3a, Supplemental Table 5). Adding plasma 2-MC to the combination of 12 clinical variables improved the mean accuracy to 0.8. The combination with the highest mean accuracy included plasma 2-MC, FSIQ, ALT, RBC, platelets, LVEF%, and cystatin C–based eGFR (mean accuracy 0.95, sensitivity 0.92, mean specificity 0.99, Supplemental Table 5). Adding plasma C3 to the combination of 12 clinical parameters had a similar effect on the mean accuracy improving it to 0.8. The combination that yielded the highest mean accuracy (0.98) included plasma C3, ALT, WBC, RBC, cystatin C–based eGFR, and height *z*-score (sensitivity 0.99 and specificity 0.99, Supplemental Table 5).

After showing that established biochemical PA parameters can improve the prediction accuracy of svmPoly models, we decided to use this approach for an unbiased identification of novel PA biomarkers. We reran svmPoly models, adding one new variable sequentially to the 12 clinical parameters. The new variables were selected from the list of 301 variables with >70% complete data, which was a cutoff selected based on a published review.^26^ In this experiment, of all 301 models, a model using plasma C3 ranked 30/301 with mean accuracy of 0.83 and the model using plasma total 2-MC ranked 23/301 with mean accuracy of 0.83.

We therefore first used the mean accuracy of 0.83 as a cutoff to identify novel biomarkers, a level that indicates greater performance than for established diagnostic biomarkers. Thirty models exceeded the cutoff of 0.83 for the mean accuracy. Of these 30 combinations, one used the in vivo 60-minute 1-^13^C-propionate oxidation study^27^ and had the mean accuracy of 0.84 (ranked 20/301, Fig. 3a). A model utilizing in vivo 60-minute 1-^13^C-propionate oxidation with the highest mean accuracy (0.96) included five variables (1-^13^C-propionate oxidation, WBC, RBC, height *z*-score, and total protein intake, Supplemental Table 5).

In our analysis, we noted that models with the highest accuracy did not utilize all features available for modeling. It prompted us to analyze the relationship between model performance and number of variables in each combination. Thus, we found that the overall performance of models which used subsets of 12 clinical parameters plus 1-^13^C-propionate oxidation peaked around 6–10 variables per combination with a narrow range of mean accuracies (Fig. 3b). This narrowing of the accuracy range suggested that some parameters may have larger influence (measured using the model output “variable importance”) than others in each model. For the model including 12 clinical parameters plus 1-^13^C-propionate oxidation, the three parameters with the highest importance were 1-^13^C-propionate oxidation (variable importance = 95.7), FSIQ (81.1), and height *z*-score (78.9) (Fig. 3c).

Finally, we performed a longitudinal study to evaluate the effect of the order and the number of study participants required to achieve the mean accuracy of ~0.85 (Fig. 3d). We found that the mean accuracy plateaued after the enrollment of the first 10–15 participants. This suggests that even in a rare disease like PA, the svmPoly modeling could extract relevant features with as few as a dozen participants.

### 1-^13^C-propionate oxidation is associated with PA outcomes

**Fig. 4:**
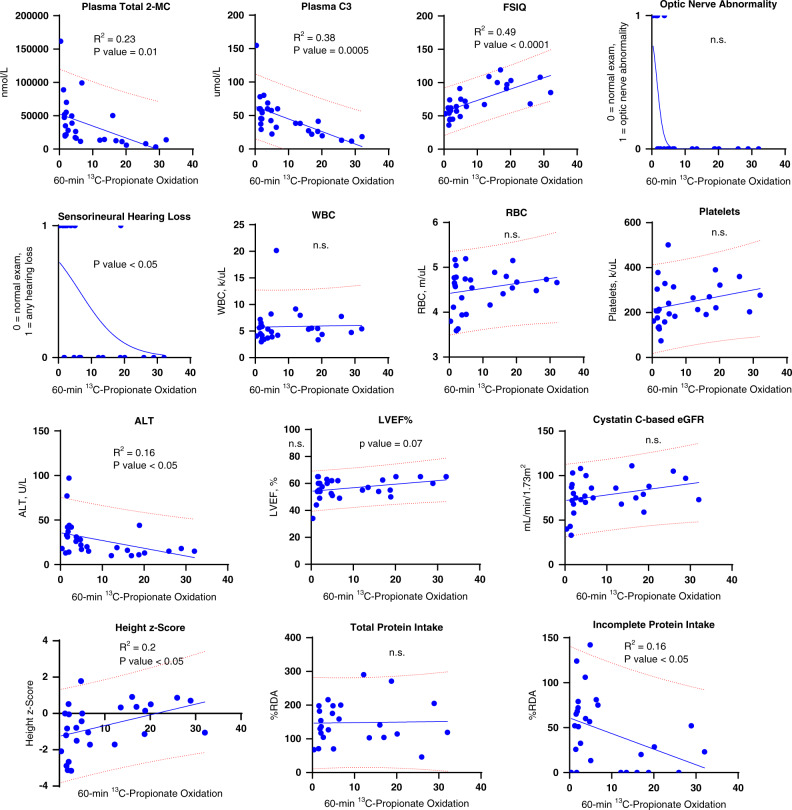
1-^13^C-Propionate oxidation is associated with plasma propionylcarnitine, total 2-methylcitrate, and select clinical parameters. In this cohort, 1-^13^C-propionate oxidation was not associated with a cystatin C–based estimated glomerular filtration rate and there was a trend toward statistical significance for LVEF% (*p* value = 0.07). Logistic regression was performed to evaluate the association between sensorineural hearing loss, optic nerve abnormality, and the 1-^13^C-propionate oxidation. ALT alanine aminotransferase, eGFR estimated glomerular filtration rate, LVEF% left ventricular ejection fraction percent, RBC red blood cell count, RDA recommended dietary allowance, WBC white blood cell count, n.s. not significant. Red dotted lines denote a 95% prediction band.

After identifying the in vivo 60-minute 1-^13^C-propionate oxidation as one of most impactful variables associated with a PA class defined by judges, we proceeded to validate its association with the 12 clinical parameters in the PA model and 2 biochemical parameters (Fig. 4). 1-^13^C-propionate oxidation correlated with plasma total 2-MC, plasma C3, FSIQ, hearing loss, ALT, height *z*-score, and the incomplete protein intake as a percent of RDA. 1-^13^C-propionate oxidation showed a favorable sensitivity and specificity in identifying cases compared to unaffected controls and good intrasubject reproducibility shown in Supplemental Fig. 3a–c.

### Validation analysis of PA biomarkers using a liver transplant subgroup

**Fig. 5:**
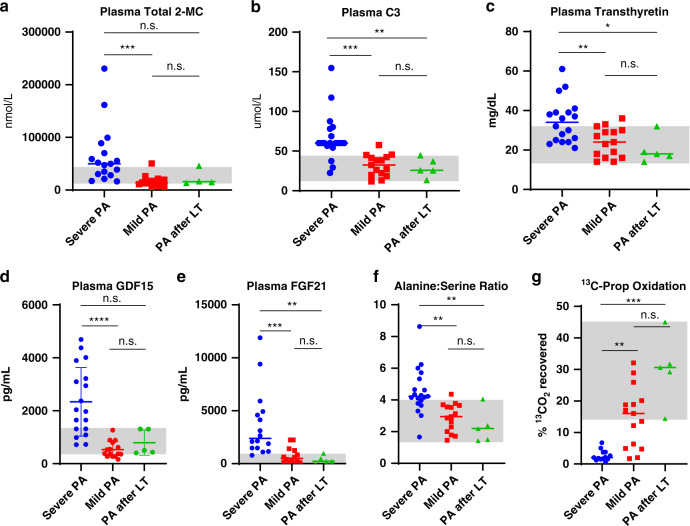
External validation analysis of novel biomarkers using data from liver-transplanted participants supported the role of select biomarkers in defining mild and severe propionic acidemia (PA). (**a**,**b**) Two known diagnostic PA biomarkers, plasma total 2-methylcitrate and propionylcarnitine, were not different between the mild PA and liver-transplanted participants. (**c**) Plasma transthyretin was ranked 42/301 by the svmPoly model. The difference between severe and mild PA as well as severe PA and PA after LT was statistically significant, but there was an overlap in values between severe PA and PA after LT. (**d**) Plasma GDF15 was significantly lower in the mild PA compared to severe PA and there was an overlap in values between severe PA and PA after LT. (**e**). Plasma FGF21 was significantly lower in mild PA and PA after LT when compared to the severe PA. There was a minimal overlap in the values between the severe PA and PA after LT classes. (**f**) In this cohort, plasma alanine:serine ratio was ranked high by the svmPoly model (8/301). However, there was a significant overlap between severe PA and PA after LT. (**g**) The mean 1-^13^C-propionate oxidation in transplanted PA participants was significantly higher that the severe PA class. Importantly, there was no overlap in values between severe PA participants and PA after LT. 2-MC total 2-methylcitrate, C3 propionylcarnitine, LT liver transplant, n.s. not significant, SVM support vector machines. Gray shading denotes the range of parameters found in transplanted PA patients. Significant *p* values are denoted by *p < 0.05, **p < 0.01, ***p < 0.001, ****p < 0.0001.

We hypothesized that liver transplantation was more likely to be offered to severely affected PA patients and that after transplantation, participants would manifest clinical and biochemical traits of milder PA. To test this hypothesis, we compared values of established and novel biomarkers among severe PA, mild PA, and PA after liver transplant groups. Plasma total 2-MC levels in liver-transplanted participants were not different from either severe or mild PA (Fig. 5a). Plasma C3 in liver-transplanted participants was lower than in severe (*p* < 0.01) but similar to mild PA participants, thus supporting our hypothesis that inherent PA severity can affect the performance of C3 (Fig. 5b).

Plasma transthyretin (prealbumin, ranked 42/301), a biomarker reported to track with renal dysfunction,^28^ was higher in severe PA than in either the mild PA group (*p* < 0.01) or PA after liver transplant (*p* < 0.05, Fig. 5c). Plasma GDF15, elevated in patients with mitochondrial disorders,^29^ was ranked only 40/301 based on the mean accuracy of 0.83 as a cutoff to identify novel biomarkers. Although GDF15 differentiated between severe and mild PA, the sample was underpowered to detect the difference in liver-transplanted participants (Fig. 5d). The potential utility of plasma transthyretin, alanine:serine ratio, and GDF15 was limited by the significant overlap of values among severe, mild, and liver-transplanted participants. FGF21 ranked 27/301, was significantly lower in the mild and transplanted PA participants (*p* < 0.001 and *p* < 0.01, respectively), and showed minimal overlap between transplanted and severe PA cases (Fig. 5e). The alanine:serine ratio, which may reflect mitochondrial dysfunction,^30^ ranked 8/301 by the svmPoly model. The plasma alanine:serine ratio was significantly higher in the severe PA than mild (*p* < 0.01) and liver-transplanted (*p* < 0.01) participants (Fig. 5f). Finally, in vivo 1-^13^C-propionate oxidation in the transplanted cohort, ranked 20/301, was significantly higher than severe (*p* < 0.001), but no different from mild PA (Fig. 5g). Of note, it was the only examined biomarker, whose values did not overlap between the severe PA group and liver-transplanted participants.

## DISCUSSION

The discovery and validation of biomarkers for inborn errors of metabolism have been a challenge due to low incidence, high mortality, medical fragility, ascertainment bias of natural history studies, and interlaboratory variability of biomarker levels.^31^ A combination of these factors likely underlies the lack of FDA-accepted validated biomarkers to support new drug and biologics applications for many inborn errors of metabolism, including PA. We aimed to address the need for pharmacodynamic biomarkers and and surrogate endpoints to facilitate clinical trials in this patient population, by extending known diagnostic biomarkers, such as plasma 2-methylcitrate and propionylcarnitine, and integrating them with novel clinical and laboratory parameters such as in vivo 1-^13^C-propionate oxidation, and plasma FGF21 and GDF15.

We provide evidence that a polynomial kernel SVM approach can extract relevant features of parsimonious PA models from a dataset with relatively few participants and with many variables (*p* >> *n*). We believe that that this discovery paradigm can be extended to other rare disease populations. To minimize the problem of overfitting, we supplemented our procedure by performing random repartitioning of the PA dataset into training and test subsets (*n* = 1,000). The drawback of this approach was the need for significant computational resources as the number of possible combinations of variables follows an exponential curve (e.g., ~8,192,000 SVM models per one subset composed of 13 variables). We were able to address this limitation by deploying SVM on the local high-performance parallel computing cluster (Biowulf, https://hpc.nih.gov, Supplemental Fig. 2).

The fact that SVM prioritized known diagnostic biomarkers, plasma total 2-methylcitrate and propionylcarnitine, in the top tenth percentile is not surprising. However, the utility of these biomarkers alone to classify PA participants was adversely affected by CKD (Fig. 1h, i), a comorbidity to be considered when selecting PA patients for future clinical trials.^28^ The association of the total 2-methylcitrate and propionylcarnitine with renal function prompted us to search for novel biomarkers less affected by CKD. To this end, we selectively reviewed other variables in the top 20th percentile of the ranking order. Among potential biomarkers prioritized by the svmPoly model were plasma transthyretin (ranked 42/301), GDF15 (40/301), FGF21 (27/301), and alanine:serine ratio (8/301). Although transthyretin (prealbumin) has been proposed to serve as an integrated index to assess protein intake in organic acidemias,^32^ our previous data revealed a strong association with renal and cardiac function in PA.^28^ The ability of alanine:serine ratio to improve the accuracy of svmPoly model was an unexpected finding; however, it fits well with the role of alanine in reflecting impaired oxidation of pyruvate and deficient synthesis of serine, both seen in mitochondrial disorders.^30^ GDF15 and FGF21 have been previously proposed as potential biomarkers in propionic acidemia and *MMUT*-related methylmalonic acidemia.^29,33–35^ In this study, FGF21 values had minimal overlap among severe, mild, and liver-transplanted PA subjects (Fig. 5e). Transthyretin, GDF15, FGF21, and alanine:serine ratio showed dependence on the cystatin C–based and creatinine-based eGFR (data not shown) and thus their potential utility in clinical trials may not extend to patients with, or at high risk of, advanced CKD. Finally, in vivo 1-^13^C-propionate oxidation, ranked 20/301, clearly separated liver-transplanted participants from severely affected PA patients, and did not correlate with cystatin C–based eGFR. This suggests that in vivo propionate oxidation may be linked to the residual activity of propionyl-CoA carboxylase activity and has the potential to perform well in clinical assessment of gene^12^ or mRNA therapies for propionic acidemia and *MMUT*-related methylmalonic acidemia^27,36,37^

Limitations of our approach stem from the limited size and the cross-sectional nature of the datasets. First, the liver-transplanted subgroup is small, and as a result, our ability to isolate the effects of immunosuppression on response biomarkers might be limited. While liver-transplanted patients may acquire some traits of mild PA, select organs (e.g., brain, heart, and kidney) are still at risk for long-term complications even after transplant. Whole-body propionate metabolism in transplanted patient is likely distinct from mild PA individuals whose minimal residual activity of PCC may result in tissue-specific protective effects. Therefore, this work will be most relevant to those future clinical trials that aim to utilize response biomarkers in the context of novel therapeutics aiming to restore propionate oxidation in the liver. Second, our enrollment efforts cannot fully capture the effects of antecedent events on the outcomes of PA. For example, some patients may have undergone extensive changes in the diet before they underwent studies via our protocol, thus diminishing our ability to detect the effects of medical foods on growth outcomes. Finally, this study represents only a proof-of-principle approach awaiting validation of this machine learning approach to extracting biomarkers in other rare disease datasets.

In summary, we have demonstrated that subject experts’ knowledge supplemented by machine learning can evaluate severity biomarkers in PA. This discovery paradigm could be extended to other rare disorders, where disease classification and discovery of biomarkers are needed but few research subjects are likely to be available.

## Supplementary information

Supplemental Table 5

Supplemental Tables

Supplementary Figure 1

Supplementary Figure 2

Supplementary Figure 3

Supplementary Figure 4

Supplemental Materials

## Data Availability

Any materials, data, and datasets produced from or used for this study will be made available upon request.
