# Supplementary material for: Severity modeling of propionic acidemia using clinical and laboratory biomarkers
Source: Genet Med. 2021 May 18;23(8):1534–42. doi: 10.1038/s41436-021-01173-2 (PMC8354856; doi:10.1038/s41436-021-01173-2)
Supplement: Supplementary file 2 — Supplemental Tables [file 41436_2021_1173_MOESM2_ESM.docx]

**Supplemental Table 1.** *Select clinical and laboratory characteristics of study participants.*

| N | Transplant Status | Gene Affected | Variant 1 (cDNA) | Variant 1 (protein) | Variant 2 (cDNA) | Variant 2 (protein) | Total 2-MC, nM | C3, uM | 60 min 1-^13^C-Propionate |
| --- | --- | --- | --- | --- | --- | --- | --- | --- | --- |
| 1 | NT | PCCA | c.722del | p.Gly241ValfsTer19 | c.722del | p.Gly241ValfsTer19 | n.a. | 60.0 | n.a. |
| 2 | NT | PCCA | ex 23 and 24 deletion | n.a. | ex 23 and 24 deletion | n.a. | 38906.0 | 68.8 | 3.8 |
| 3 | NT | PCCA | c.802C>T | p.Arg268Cys | c.1899+4_1899+7delAGTA | Intronic | 3167.0 | 11.7 | 28.9 |
| 4 | NT | PCCA | c.1268C>T | p.Pro423Leu | c.1899+4_1899+7delAGTA | Intronic | 11568.0 | 32.4 | 6.3 |
| 5 | LT | PCCA | c.1288C>T | p.Arg430Ter | c.776T>G | p.Leu259Arg | 7768.0 | 13.5 | 29.2 |
| 6 | NT | PCCA | c.742G>A | p.Glu248Lys | c.1430G>T | p. Gly477Val | 12567.0 | 22.1 | 17.0 |
| 7 | NT | PCCA | c.716+5G>C | Intronic | c.782A>G | p.Glu261Gly | 16251.0 | 22.4 | 5.0 |
| 8 | NT | PCCA | c.1572_1573del | p.Gln524HisfsTer29 | c.434T>C | p.Phe145Ser | 8007.0 | 13.1 | 25.9 |
| 9 | NT | PCCA | c.600+1G>A | Canonical splice | c.2119-9A>G | Intronic | 161679.0 | 154.7 | 0.4 |
| 10 | NT | PCCA | c.1284+1G>A | Canonical splice | c.1684T>C | p.Ser562Pro | 11221.0 | 26.2 | 18.8 |
| 11 | NT | PCCA | c.1284+1G>A | Canonical splice | c.1684T>C | p.Ser562Pro | 13395.0 | 38.3 | 12.2 |
| 12 | NT | PCCA | c.893A>G | p.Lys298Arg | ex 13-20 deletion | n.a. | 26457.0 | 57.5 | 4.9 |
| 13 | NT | PCCA | c.1284+1G>A | Canonical splice | c.2027del | p.Lys676SerfsTer6 | 19608.0 | 45.6 | 1.7 |
| 14 | LT | PCCA | c.782A>G | p.Glu261Gly | c.923dup | p.Leu308PhefsTer35 | 16675.0 | 44.7 | 45.0 |
| 15 | NT | PCCA | c.866_867del | p.Glu289ValfsTer53 | c.2162_2163insAG | p.Asp722GlyfsTer32 | 88864.0 | >60 | 1.2 |
| 16 | NT | PCCA | c.862A>G | p.Arg288Gly | c.1214C>G | p.Pro405Arg | 21553.0 | 41.3 | 18.9 |
| 17 | LT | PCCA | c.1540+1G>C | Canonical splice | c.782A>G | p.Glu261Gly | 12408.0 | 25.8 | 30.6 |
| 18 | NT | PCCB | TBD | TBD | TBD | TBD | 230675.0 | 87.5 | n.a. |
| 19 | NT | PCCB | c.1218_1231delins TAGAGCACAGGA | p.Gly407ArgfsTer14 | c.1218_1231delins TAGAGCACAGGA | p.Gly407ArgfsTer14 | 28002.0 | 80.2 | 2.6 |
| 20 | NT | PCCB | c.386_387delinsAAC | p.Phe129Ter | c.1552del | p.Asp518ThrfsTer33 | 41924.0 | >60 | 3.6 |
| 21 | NT | PCCB | c.1218_1231delins TAGAGCACAGGA | p.Gly407ArgfsTer14 | c.990dup | p.Glu331Ter | 34916.0 | 29.3 | 1.8 |
| 22 | KT | PCCB | c.1142dup | p.Cys381TrpfsTer2 | c.1606A>G | p.Asn536Asp | 48880.0 | 18.3 | 9.1 |
| 23 | NT | PCCB | c.734G>A | p.Gly245Asp | ex 9 deletion | n.a. | 20541.0 | 40.1 | 2.5 |
| 24 | NT | PCCB | c.1218_1231delins TAGAGCACAGGA | p.Gly407ArgfsTer14 | c.1495C>T | p.Arg499Ter | 70142.0 | 54.3 | 2.1 |
| 25 | NT | PCCB | c.683C>T | p.Pro228Leu | c.1218_1231delins TAGAGCACAGGA | p.Gly407ArgfsTer14 | 5853.0 | 19.7 | 20.1 |
| 26 | NT | PCCB | c.1204del | p.Ala402HisfsTer41 | c.335G>A | p.Gly112Asp | 47361.0 | 78.0 | 1.5 |
| 27 | NT | PCCB | c.1172_1173del | p.Phe391CysfsTer2 | c.1172_1173del | p.Phe391CysfsTer2 | 55257.0 | 56.6 | 2.0 |
| 28 | NT | PCCB | c.683C>T | p.Pro228Leu | c.1218_1231delins TAGAGCACAGGA | p.Gly407ArgfsTer14 | 14435.0 | 38.2 | 13.5 |
| 29 | LT | PCCB | c.1260dup | p.Glu421Ter | c.1260dup | p.Glu421Ter | 13660.0 | 25.5 | 31.7 |
| 30 | NT | PCCB | c.337C>T | p.Arg113Ter | c.1225_1227del | p.Ile409del | 51869.0 | >60 | 1.6 |
| 31 | NT | PCCB | c.764-2del | Canonical splice | c.975_977del | p.Asp325del | 17013.0 | >60 | n.a. |
| 32 | NT | PCCB | c.764-2del | Canonical splice | c.975_977del | p.Asp325del | 30252.0 | >60 | n.a. |
| 33 | NT | PCCB | c.386_387delinsAAC | p.Phe129Ter | c.1606A>G | p.Asn536Asp | 17446.0 | 42.4 | 4.8 |
| 34 | NT | PCCB | c.1218_1231delins TAGAGCACAGGA | p.Gly407ArgfsTer14 | c.1606A>G | p.Asn536Asp | 99283.0 | >60 | 6.7 |
| 35 | NT | PCCB | c.76dup | p.Arg26ProfsTer11 | c.1218_1231delins  TAGAGCACAGGA | p.Gly407ArgfsTer14 | 49810.0 | 60.1 | 3.7 |
| 36 | NT | PCCB | c.1606A>G | p.Asn536Asp | c.1606A>G | p.Asn536Asp | 13945.0 | 18.4 | 32.0 |
| 37 | NT | PCCB | c.990dup | p.Glu331Ter | c.1225_1227del | p.Ile409del | 21000.0 | 37.4 | 1.7 |
| 38 | NT | PCCB | c.1218_1231delins TAGAGCACAGGA | p.Gly407ArgfsTer14 | c.967G>T | p.Val323Phe | 50257.0 | 27.3 | 16.0 |
| 39 | LT | PCCB | c.337C>T | p.Arg113Ter | c.1606A>G | p.Asn536Asp | 46168.0 | 37.1 | 14.4 |
| 40 | NT | TBD | TBD | TBD | TBD | TBD | 21630.0 | 45.0 | 2.0 |

**Supplemental Table 1. Abbreviations**. 2-MC – plasma total 2-methylcitrate, C3 – plasma propionylcarnitine, ex – exon, KT – kidney transplanted, LT – liver transplanted, n.a. – not available, NT – not transplanted, TBD – to be determined. *PCCA* NCBI Reference Sequence: NM_000282.3. *PCCB* NCBI Reference Sequence: NM_000532.4.

**Analysis of Missingness Completely at Random (MCAR)**

**Supplemental Table 2A.** *Clinical parameters and biomarkers are not associated with the missingness in FSIQ*

| **Missing data analysis using FSIQ as a dependent variable** | **Continuous vs categorical** | **FSIQ not missing** | **FSIQ missing** | ***p*-value** |
| --- | --- | --- | --- | --- |
| Plasma propionylcarnitine (C3), umol/L | Mean (SD) | 51.0 (30.3) | 57.2 (4.0) | 0.778 |
| Plasma total 2-methylcitrate, nmol/L | Mean (SD) | 40939.3 (47241.7) | 70142.0 (NA) | 0.547 |
| 1-^13^C-propionate oxidation, % recovery | Mean (SD) | 8.7 (8.5) | 2.1 (NA) | 0.454 |
| Optic nerve abnormality | Absent = 0 (%) | 20 (95.2) | 1 (4.8) | 1.000 |
|  | Present = 1 (%) | 8 (100.0) | 0 (0.0) |  |
| ALT, U/L | Mean (SD) | 28.7 (20.0) | 41.5 (3.5) | 0.379 |
| White blood cell count, k/uL | Mean (SD) | 5.7 (3.1) | 3.2 (0.5) | 0.265 |
| Red blood cell count, m/uL | Mean (SD) | 4.5 (0.5) | 4.2 (0.9) | 0.466 |
| Platelet, k/uL | Mean (SD) | 243.3 (92.8) | 151.0 (33.9) | 0.176 |
| Left ventricular ejection fraction, % | Mean (SD) | 56.2 (9.1) | 54.0 (NA) | 0.812 |
| Cystatin C-based eGFR | Mean (SD) | 74.8 (21.2) | 58.0 (NA) | 0.440 |
| Height, z-score | Mean (SD) | -0.8 (1.4) | -0.9 (1.2) | 0.957 |
| Total protein intake, % of RDA | Mean (SD) | 146.2 (57.6) | 124.2 (17.2) | 0.600 |
| Incomplete protein intake, % of RDA | Mean (SD) | 41.5 (38.9) | 53.5 (3.5) | 0.671 |

**Supplemental Table 2B.** *Clinical parameters and biomarkers are not associated with the missingness of 1-^13^C-propionate oxidation*

| **Missing data analysis using 1-^13^C-propionate oxidation as a dependent variable** | **Continuous vs categorical** | **Oxidation not missing** | **Oxidation missing** | ***p*-value** |
| --- | --- | --- | --- | --- |
| Plasma propionylcarnitine (C3), umol/L | Mean (SD) | 47.7 (30.1) | 60.2 (27.2) | 0.263 |
| Plasma total 2-methylcitrate, nmol/L | Mean (SD) | 37424.8 (36907.9) | 53555.9 (67987.5) | 0.386 |
| Full scale intellectual quotient (FSIQ) | Mean (SD) | 71.3 (23.9) | 64.4 (17.2) | 0.441 |
| Optic nerve abnormality | Absent = 0 (%) | 16 (76.2) | 5 (23.8) | 1.000 |
|  | Present = 1 (%) | 6 (75.0) | 2 (25.0) |  |
| ALT, U/L | Mean (SD) | 26.0 (19.0) | 38.7 (19.4) | 0.100 |
| White blood cell count, k/uL | Mean (SD) | 6.0 (3.4) | 4.4 (1.2) | 0.174 |
| Red blood cell count, m/uL | Mean (SD) | 4.5 (0.5) | 4.4 (0.7) | 0.467 |
| Platelet, k/uL | Mean (SD) | 235.9 (99.1) | 242.5 (80.4) | 0.854 |
| Sensorineural hearing loss | Absent = 0 (%) | 11 (78.6) | 3 (21.4) | 1.000 |
|  | Present = 1 (%) | 10 (76.9) | 3 (23.1) |  |
| Left ventricular ejection fraction, % | Mean (SD) | 55.3 (7.1) | 58.4 (13.1) | 0.389 |
| Cystatin C-based eGFR | Mean (SD) | 77.0 (20.0) | 67.2 (23.4) | 0.242 |
| Height, z-score | Mean (SD) | -0.6 (1.3) | -1.2 (1.3) | 0.244 |
| Total protein intake, % of RDA | Mean (SD) | 144.4 (61.6) | 145.7 (39.2) | 0.955 |
| Incomplete protein intake, % of RDA | Mean (SD) | 45.1 (39.2) | 34.6 (34.7) | 0.484 |

**Abbreviations.** ALT – alanine aminotransferase, C3 – plasma propionylcarnitine, eGFR – estimated glomerular filtration rate, FSIQ – full scale IQ, NA – not applicable, RDA – recommended daily allowance, SD – standard deviation, U/L – units per liter.

**Interrater Agreement**

**Supplemental Table 3.** *Assessment of the interrater agreement*.

| **Participant** | **Rater 1** | **Rater 2** | **Rater 3** | **Agreement** |
| --- | --- | --- | --- | --- |
| 1 | 1 | 1 | 1 | 1 |
| 2 | 1 | 2 | 1 | 0.67 |
| 3 | 1 | 1 | 1 | 1 |
| 4 | 2 | 1 | 1 | 0.67 |
| 5 | 1 | 1 | 1 | 1 |
| 6 | 2 | 2 | 2 | 1 |
| 7 | 2 | 2 | 2 | 1 |
| 8 | 1 | 1 | 1 | 1 |
| 9 | 2 | 2 | 2 | 1 |
| 10 | 1 | 1 | 1 | 1 |
| 11 | 1 | 1 | 1 | 1 |
| 12 | 2 | 2 | 2 | 1 |
| 13 | 2 | 1 | 1 | 0.67 |
| 14 | 1 | 1 | 1 | 1 |
| 15 | 2 | 2 | 2 | 1 |
| 16 | 1 | 1 | 1 | 1 |
| 17 | 2 | 2 | 1 | 0.67 |
| 18 | 1 | 1 | 1 | 1 |
| 19 | 2 | 2 | 2 | 1 |
| 20 | 2 | 2 | 2 | 1 |
| 21 | 2 | 2 | 2 | 1 |
| 22 | 1 | 1 | 2 | 0.67 |
| 23 | 2 | 2 | 1 | 0.67 |
| 24 | 2 | 2 | 1 | 0.67 |
| 25 | 1 | 1 | 1 | 1 |
| 26 | 1 | 1 | 1 | 1 |
| 27 | 2 | 2 | 2 | 1 |
| 28 | 1 | 1 | 1 | 1 |
| 29 | 1 | 1 | 2 | 0.67 |
| 30 | 1 | 2 | 1 | 0.67 |
| 31 | 2 | 2 | 2 | 1 |
| 32 | 1 | 1 | 1 | 1 |
| 33 | 2 | 2 | 2 | 1 |
| 34 | 2 | 1 | 2 | 0.67 |
| # of unlike responses | 2 | 3 | 5 |  |
| Average Interrater Reliability | | | | 0.9 |

The average interrater reliability was 0.9, Fleiss kappa for *m* number of raters was 0.605 (*p* < 0.0001). 1 = severe PA, 2 = mild PA.

**Supplemental Table 4**. *Classification of propionic acidemia assigned by judges revealed tractable clinical parameters associated with “mild” and “severe” classes of propionic acidemia.*

| **Clinical Parameters** | **Estimate** | **SD** | **t value** | **P value** |
| --- | --- | --- | --- | --- |
| Sex | -0.0069 | 0.176 | -0.039 | 0.969 |
| Age | -0.0105 | 0.008 | -1.237 | 0.225 |
| Gene affected (*PCCA* or *PCCB*) * | **-0.3797** | **0.166** | **-2.285** | **0.029** |
| Full Scale IQ (FSIQ) *** | **0.0143** | **0.003** | **4.389** | **0.0001** |
| Optic nerve abnormality* | **-0.4464** | **0.196** | **-2.276** | **0.031** |
| Alanine aminotransferase** | **-0.0126** | **0.004** | **-3.122** | **0.004** |
| White blood cell count* | **0.065** | **0.027** | **2.393** | **0.0227** |
| Red blood cell count** | **0.516** | **0.147** | **3.515** | **0.0013** |
| Platelet count* | **0.0019** | **0.001** | **2.109** | **0.0429** |
| Sensorineural hearing loss* | **-0.4835** | **0.175** | **-2.762** | **0.0106** |
| Left ventricular ejection fraction, % | 0.0118 | 0.01 | 1.184 | 0.245 |
| Cystatin C-based eGFR* | **0.009** | **0.004** | **2.29** | **0.029** |
| Height z-score*** | **0.209** | **0.056** | **3.743** | **0.0007** |
| Total protein intake as a % of RDA | 0.003 | 0.002 | 1.588 | 0.124 |
| Incomplete protein intake as a % of RDA | 0.0002 | 0.002 | 0.104 | 0.918 |
| Plasma total 2-methylcitrate (2-MC)** | **-5.31e-06** | **1.68e-06** | **-3.165** | **0.004** |
| Plasma propionylcarnitine (C3)*** | **-0.01** | **0.002434** | **-4.189** | **0.0002** |

Results of the logistic model using judges scores (“severe” PA = 0, “mild” PA = 1) against a given clinical parameter. **Abbreviations.** 2-MC – total 2-methylcitrate, C3 – propionylcarnitine, eGFR – estimated glomerular filtration rate, PA – propionic acidemia, RDA – recommended daily allowance, SD – standard deviation. Significant *p-*values are highlighted in bold and associated clinical parameters are denoted by * < 0.05, ** < 0.01, *** < 0.001, **** < 0.0001.
