# Supplementary figures and images for: Severity modeling of propionic acidemia using clinical and laboratory biomarkers
Source: Genet Med. 2021 May 18;23(8):1534–42. doi: 10.1038/s41436-021-01173-2 (PMC8354856; doi:10.1038/s41436-021-01173-2)

# Supplemental Figure 1

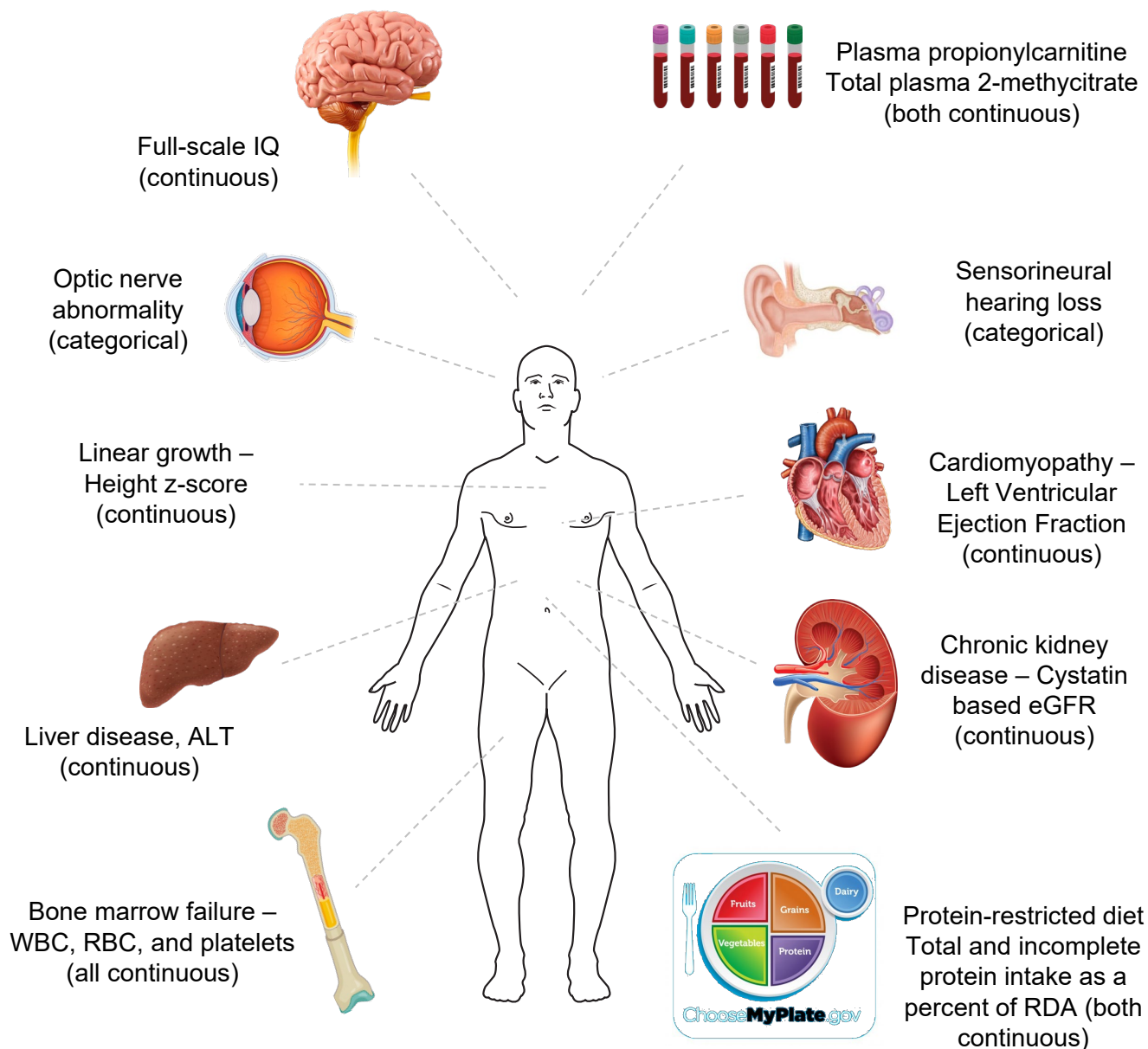

Supplement: Supplementary file 3 — Supplementary Figure 1 [file 41436_2021_1173_MOESM3_ESM.pdf]

## Supplemental Figure 2

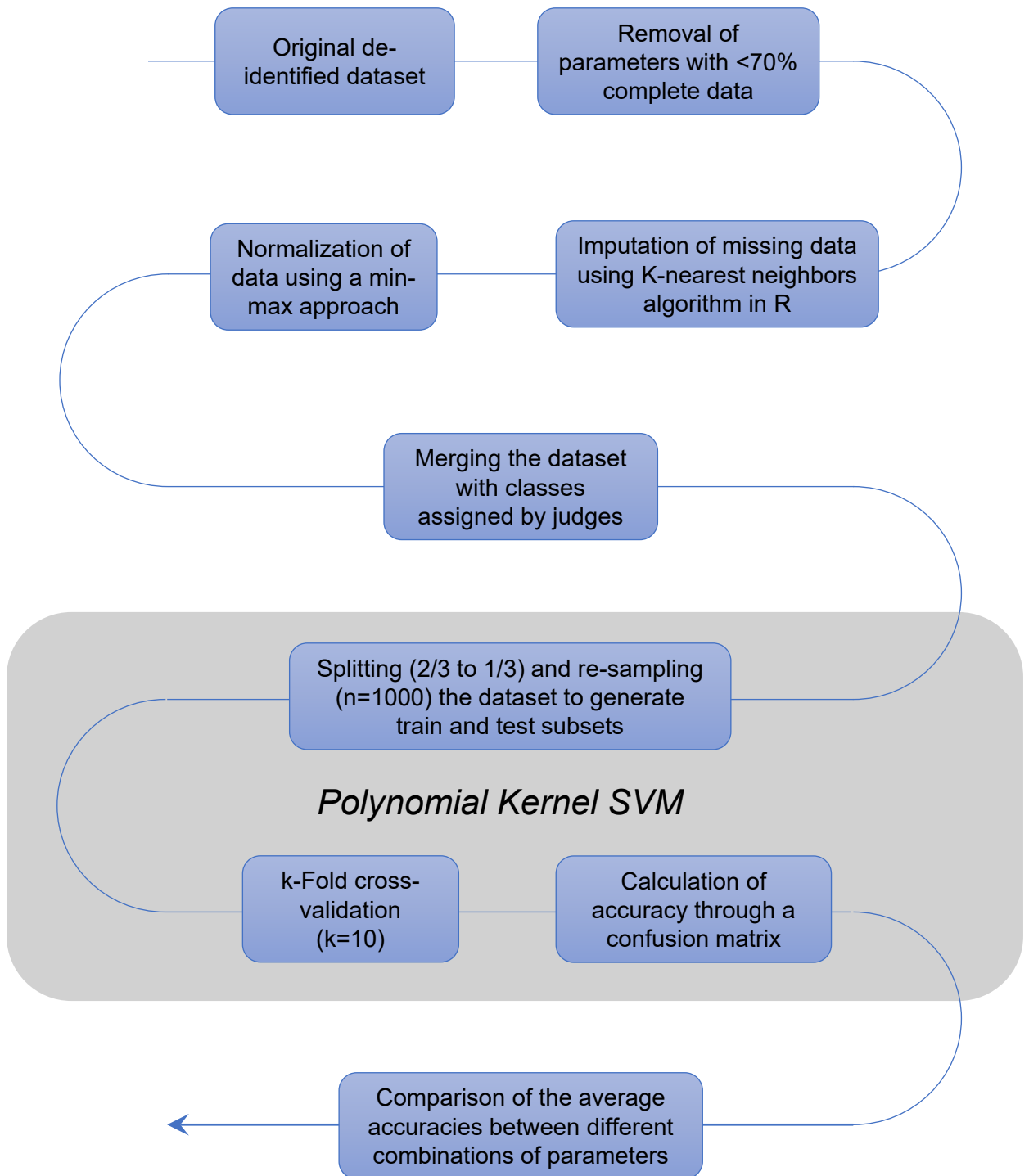

Supplement: Supplementary file 4 — Supplementary Figure 2 [file 41436_2021_1173_MOESM4_ESM.pdf]

# Supplemental Figure 3

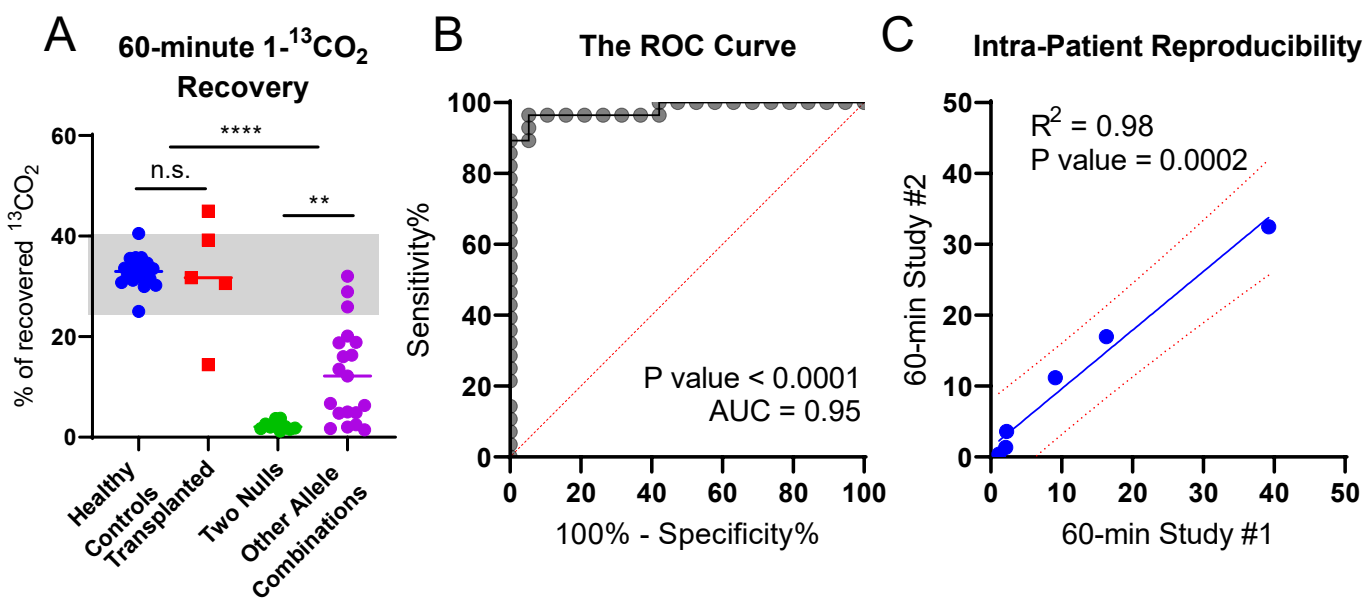

Supplement: Supplementary file 5 — Supplementary Figure 3 [file 41436_2021_1173_MOESM5_ESM.pdf]

# Supplemental Figure 4

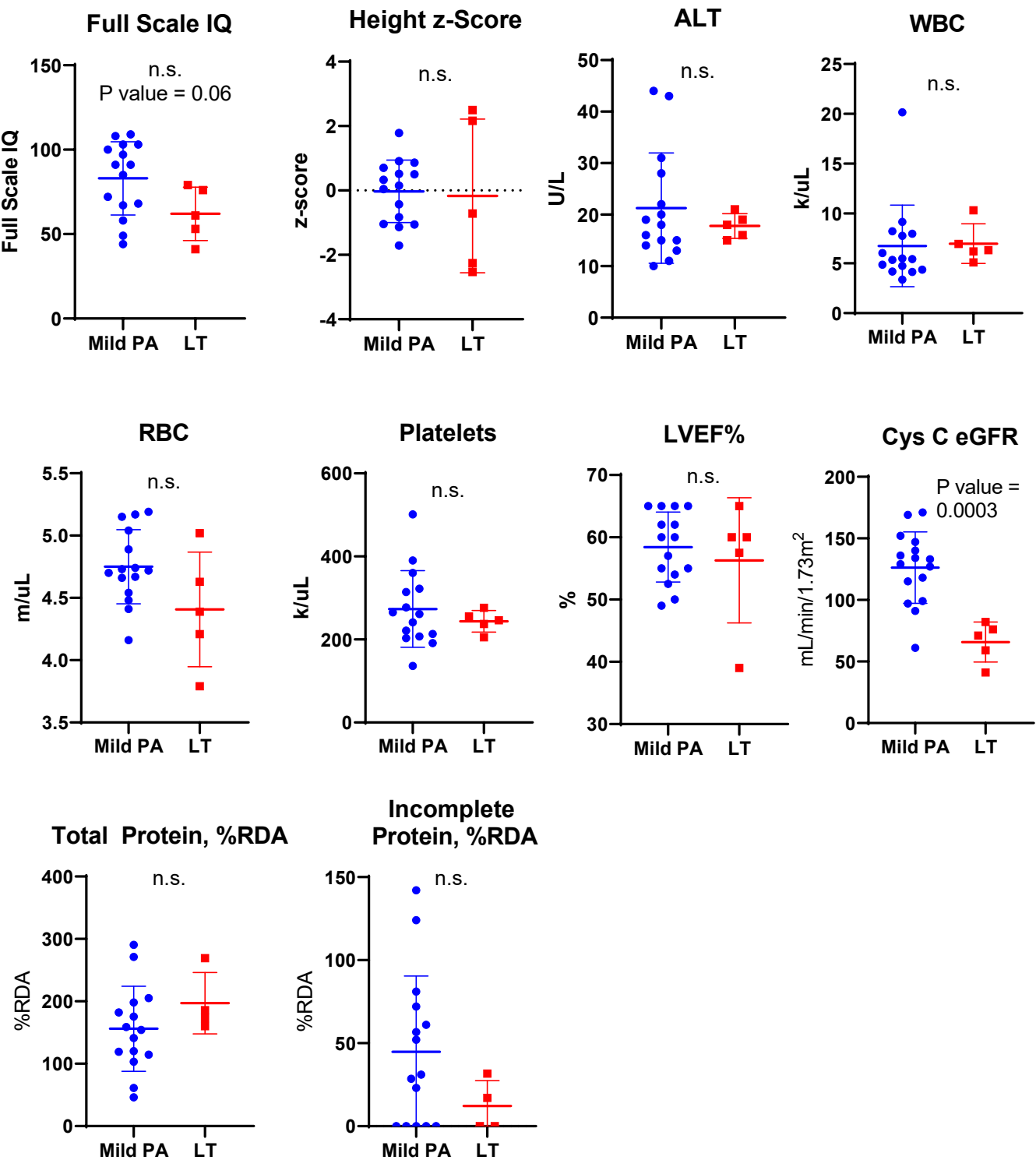

Supplement: Supplementary file 6 — Supplementary Figure 4 [file 41436_2021_1173_MOESM6_ESM.pdf]
