# Supplementary material for: Severity modeling of propionic acidemia using clinical and laboratory biomarkers
Source: Genet Med. 2021 May 18;23(8):1534–42. doi: 10.1038/s41436-021-01173-2 (PMC8354856; doi:10.1038/s41436-021-01173-2)
Supplement: Supplementary file 7 — Supplemental Materials [file 41436_2021_1173_MOESM7_ESM.docx]

**Supplemental Materials and Methods**

*Patient cohort and clinical protocols*

Participants or legal guardians provided written informed consent. All 40 participants with PA, aged 2–53 years, were evaluated at the NIH Clinical Research Center. The dataset was supplemented with selected results (e.g. genotype) obtained through clinical records from outside hospitals or other NIH protocols (ClinicalTrials.gov identifiers: NCT00369421 and NCT01780168).

Dietary intake data were collected using subject-reported three-day food records and analyzed using Nutrition Data System for Research software developed by the Nutrition Coordinating Center (University of Minnesota, Minneapolis, MN). Nutritional analysis of metabolic formulas was performed using MetabolicPro nutrient analysis software program developed by Genetic Metabolic Dietitians International (Hillsborough, NC).

In participants age <19 years, estimated glomerular filtration rate (eGFR) was calculated using the updated creatinine-based Schwartz bedside equation (2009)^1^ and cystatin C-based Schwartz equation (2012).^2^ In participants > 19 years, eGFR was calculated using the Chronic Kidney Disease Epidemiology Collaboration (CKD-EPI) Creatinine Equation (2009),^3^ and the CKD-EPI Cystatin C Equation (2012).^4^

Biochemical testing relevant to PA was performed in all 40 participants. In 6/40 participants, the reference lab reported plasma propionylcarnitine levels as “> 60 umol/L”, which were entered in the dataset as 60 umol/L. Plasma total 2-methylcitrate levels prior to liver transplantation were not available. Human fibroblast growth factor 21 (FGF21) was measured using R&D System’s Human FGF21 Quantikine ELISA (Minneapolis, MN, USA). Human growth differentiation factor 15 (GDF15) was measured either clinically by a reference lab or using R&D System’s Human GDF15 Quantikine ELISA (Minneapolis, MN, USA).

*Machine Learning*

Full scale IQ (FSIQ) or *in vivo* 1-^13^C-propionate oxidation were evaluated for the missingness-completely-at-random *via* a chi square statistic within R package MissMech (**Supplemental Tables 2A and 2B**).^5,6^ The following 14 clinical and laboratory variables were selected based on their ability to comprehensively describe the biochemical severity and involvement of organs affected by PA: plasma C3 (umol/L), plasma total 2-MC (nmol/L), FSIQ, optic nerve abnormality (absent = 0, present = 1), sensorineural hearing loss (absent = 0, present = 1), height z-score, alanine aminotransferase (ALT, units per liter), peripheral white blood cell count (WBC, k/uL), peripheral red blood cell count (RBC, m/uL), peripheral platelet count (k/uL), left ventricular ejection fraction (LVEF%), cystatin C-based estimated glomerular filtration rate (eGFR mL/min/1.73 m^2^), total protein intake (% of recommended dietary allowance), and incomplete protein intake (% of recommended dietary allowance). Plasma ammonia, amylase, and lipase were not included in the dataset because they were not part of the clinical research protocol and were only ordered on as needed basis.

We used a prototype of the organic acidemia severity scale, originally developed by Walter *et al.* (1989) and updated by Thompson *et al*. (1990) to benchmark the accuracy of svmPoly models generated in our experiments. We used five variables from our dataset, FSIQ, height z-score, presence of a gastrostomy tube, total protein intake as percentage of recommended dietary allowance, and frequency of hospitalization in the last two years as surrogates for “IQ”, “Height”, “Appetite”, “Protein tolerance”, and “Acute episodes of acidosis requiring IV therapy in 2 years prior to investigations”, used by Walter *et al.* (1989) and later studies in patients with methylmalonic and propionic acidemias by Thompson *et al*. (1990).^7,8^

After formatting, imputing missing data, and normalizing parameters using the procedure outlined above, data was split into training and test subsets using a 2:1 ratio. To extract a sparse model from the p >> n dataset, the dataset was randomly re-partitioned 1000 times into training and test subsets. The output results for each partition were aggregated and analyzed (the mean and standard deviation of accuracy, kappa, sensitivity, specificity, **Supplemental Table 5**).

In the k-means algorithm (R package *stats* (v3.6.2) function kmeans), we used Hartigan-Wong algorithm by default. The number of initial centers was set according the discussion as outlined above (shoulder and silhouette methods, input from the clinical judges). We set the nstart parameter (the number of random sets) as 100.

Hyperparameters for R package *caret* were set as “degree” = 1, scale = 1, c = 1. preProcess was set to “pca”, “scale”, “center”.

**Supplemental Figure Legends**

**Supplemental Figure 1**. **The definition of a propionic acidemia disease model.** Our disease model was defined using 12 clinical parameters and previously known parameters. Variable types are denoted in parentheses. This dataset did not track the levels of amylase or lipase as biomarkers of pancreatitis. Total and incomplete protein intake as percent of recommended daily allowance (RDA) were used to define dietary management.

**Supplemental Figure 2**. **Dataflow of the unbiased selection and evaluation of biomarkers using supervised machine learning.** **Abbreviations**: SVM – support vector machine.

**Supplemental Figure 3**. **1-^13^C-propionate oxidation performance in propionic acidemia participants.** Percent of recovered ^13^CO_2_ in exhaled breath collected over 120 minutes is a proxy readout of the *in vivo* oxidation of enterally administered 1-^13^C-propionate. **a**. ^13^CO_2_ recovery in liver-transplanted participants was not different from healthy volunteers. Non-transplanted PA patients had a lower recovery of ^13^CO_2_ compared to healthy volunteers (P value < 0.0001). Recovery of ^13^CO_2_ in participants harboring 2 null alleles was lower compared to participants with any other combination of alleles (e.g. one non-sense plus one missense, one non-sense plus one pathogenic intronic variant, two missense variants, etc). **b**. The ROC curve of the ^13^CO2 recovery demonstrates assay’s ability to discriminate between healthy controls and PA participants over a range of discrimination thresholds (AUC = 0.97, P value < 0.0001). **c**. Six PA participants underwent the test more than once. A comparison of % ^13^CO_2_ recovery between their first and second study revealed a strong and significant linear association supportive of the test’s intra-patient reproducibility.

**Supplemental Figure 4**. **A comparison of continuous parameters between liver transplanted PA *vs*. “mild” PA participants.** To evaluate one of our main premises that liver-transplanted patients share traits with “mild” PA participants, we performed comparative analysis of continuous parameters that were used to define the clinical model of PA: full scale IQ (FSIQ), height z-score, alanine aminotransferase (ALT, units per liter), peripheral white blood cell count (WBC, k/uL), peripheral red blood cell count (RBC, m/uL), peripheral platelet count (k/uL), left ventricular ejection fraction (LVEF%), cystatin C-based estimated glomerular filtration rate (eGFR mL/min/1.73 m^2^), total protein intake (% of recommended dietary allowance), and incomplete protein intake (% of recommended dietary allowance). Of these, cystatin C-based eGFR was statistically significantly lower in liver-transplanted participants, a phenomenon reviewed elsewhere (Shchelochkov et al, 2019). There was a trend towards significance in FSIQ. Although other clinical and laboratory parameters were not significant, we could be underpowered to detect the difference given a relatively small number of transplanted participants in this cohort.

**References Listed in the Supplemental Materials**

1. Schwartz GJ, Work DF. Measurement and estimation of GFR in children and adolescents. Clinical journal of the American Society of Nephrology : CJASN 2009;4:1832-43.

2. Schwartz GJ, Schneider MF, Maier PS, et al. Improved equations estimating GFR in children with chronic kidney disease using an immunonephelometric determination of cystatin C. Kidney international 2012;82:445-53.

3. Levey AS, Stevens LA, Schmid CH, et al. A new equation to estimate glomerular filtration rate. Annals of internal medicine 2009;150:604-12.

4. Inker LA, Schmid CH, Tighiouart H, et al. Estimating Glomerular Filtration Rate from Serum Creatinine and Cystatin C. New England Journal of Medicine 2012;367:20-9.

5. Ewen Harrison, Tom Drake, Riinu Ots. finalfit: Quickly Create Elegant Regression Results Tables and Plots when Modelling. R package version 100,2020.

6. Jamshidian M, Jalal S, Jansen C. MissMech: An R Package for Testing Homoscedasticity, Multivariate Normality, and Missing Completely at Random (MCAR). J Stat Softw 2014;56:31.

7. Thompson GN, Walter JH, Bresson JL, et al. In vivo propionate oxidation as a prognostic indicator in disorders of propionate metabolism. Eur J Pediatr 1990;149:408-11.

8. Walter JH, Michalski A, Wilson WM, Leonard JV, Barratt TM, Dillon MJ. Chronic renal failure in methylmalonic acidaemia. European Journal of Pediatrics 1989;148:344-8.
